# Pulsatile Controlled Release and Stability Evaluation of Polymeric Particles Containing *Piper nigrum* Essential Oil and Preservatives

**DOI:** 10.3390/ma15155415

**Published:** 2022-08-05

**Authors:** Sidney Gomes Azevedo, Ana Luisa Farias Rocha, Ronald Zico de Aguiar Nunes, Camila da Costa Pinto, Ştefan Ţălu, Henrique Duarte da Fonseca Filho, Jaqueline de Araújo Bezerra, Alessandra Ramos Lima, Francisco Eduardo Gontijo Guimarães, Pedro Henrique Campelo, Vanderlei Salvador Bagnato, Natalia Mayumi Inada, Edgar Aparecido Sanches

**Affiliations:** 1 Laboratory of Nanostructured Polymers (NANOPOL), Federal University of Amazonas (UFAM), Manaus 69067-005, AM, Brazil; 2 Graduate Program in Chemistry (PPGQ), Federal University of Amazonas (UFAM), Manaus 69067-005, AM, Brazil; 3 Graduate Program in Materials Science and Engineering (PPGCEM), Federal University of Amazonas (UFAM), Manaus 69067-005, AM, Brazil; 4 Graduate Program in Physics (PPGFIS), Federal University of Amazonas (UFAM), Manaus 69067-005, AM, Brazil; 5 The Directorate of Research, Development and Innovation Management (DMCDI), Technical University of Cluj-Napoca, 15 Constantin Daicoviciu St., 400020 Cluj-Napoca, Cluj County, Romania; 6 Laboratory of Nanomaterials Synthesis and Nanoscopy (LSNN), Federal University of Amazonas (UFAM), Manaus 69067-005, AM, Brazil; 7 Analytical Center, Federal Institute of Education, Science and Technology of Amazonas (IFAM), Manaus 69020-120, AM, Brazil; 8 São Carlos Institute of Physics (IFSC), University of São Paulo (USP), São Carlos 13563-120, SP, Brazil; 9 Department of Food Technology, Federal University of Viçosa (UFV), Viçosa 36570-900, MG, Brazil; 10 Hagler Institute for Advanced Studies, Texas A&M University, College Station, TX 77843-3572, USA

**Keywords:** *Piper nigrum*, polymeric particles, stability evaluation, controlled release, pulsatile mechanism

## Abstract

Considerable efforts have been spent on environmentally friendly particles for the encapsulation of essential oils. Polymeric particles were developed to encapsulate the essential oil from *Piper nigrum* based on gelatin and poly–*ε*–caprolactone (PCL) carriers. Gas Chromatography ((Flame Ionization Detection (GC/FID) and Mass Spectrometry (GC/MS)), Atomic Force Microscopy (AFM), Nanoparticle Tracking Analysis (NTA), Confocal Laser Scanning Microscopy (CLSM), Attenuated Total Reflectance–Fourier-transform Infrared Spectroscopy (ATR–FTIR), and Ultraviolet–Visible (UV–VIS) spectroscopy were used for the full colloidal system characterization. The essential oil was mainly composed of *β*-caryophyllene (~35%). The stability of the encapsulated systems was evaluated by Encapsulation Efficiency (EE%), electrical conductivity, turbidity, pH, and organoleptic properties (color and odor) after adding different preservatives. The mixture of phenoxyethanol/isotialzoni-3-one (PNE system) resulted in enhanced stability of approximately 120 and 210 days under constant handling and shelf-life tests, respectively. The developed polymeric system presented a similar controlled release in acidic, neutral, or basic pH, and the release curves suggested a pulsatile release mechanism due to a complexation of essential oil in the PCL matrix. Our results showed that the developed system has potential as an alternative stable product and as a controlling agent, due to the pronounced bioactivity of the encapsulated essential oil.

## 1. Introduction

The encapsulation technique represents a powerful tool to improve the properties of biomolecules with low stability under environment conditions [1]. Increasing interest in polymeric particles has resulted in new technological applications [2,3,4,5,6]. Controlled release of bioactive compounds is the basic principle of encapsulated systems [7]. Considerable efforts have been devoted to the development of environmentally friendly particles for encapsulation of essential oils [8,9,10,11,12,13,14,15], which have been widely applied as alternative controlling agents for agricultural pests [16,17,18] and for pharmacological purposes [19,20]. Specifically, the effectiveness of *Piper nigrum* (black pepper) essential oil against insects, mites, larvae, and aphids has been widely reported [21,22], as well as for the treatment of various diseases, such as intermittent fever, dysentery, stomachache, worms, and piles [23]. Moreover, the encapsulation of essential oils for controlled release formulations can improve their efficiency and reduce environmental damage due to their low toxicity [24,25].

Colloidal systems consisting of biodegradable carriers were developed herein, based on the addition of different preservatives. The combination of gelatin and poly-*ε*-caprolactone (PCL) represents a technological alternative for new particle designs, allowing specific release mechanisms, as well as an enhanced encapsulation efficiency and biodegradability [10,26,27,28]. However, specific stability tests are required to evaluate the behavior of a formulation as a function of time [10,29]. The use of preservatives in colloidal systems is an effective alternative to increase their shelf-life [30], especially by delaying the exposure of the bioactive compound into the medium, due to the particle destabilization. However, few reports have focused on both the use of preservatives in controlled release systems and the evaluation of the formulation stability under handling or storage [10,30]. Herein, the preservatives phenoxyethanol/methylisothiazolinone, methylisothiazolinone, sodium benzoate, thymol, and ethylenediaminetetraacetic acid were added to the developed colloidal systems.

A stability evaluation of colloidal systems constituted of polymeric particles is not simple to perform, because different parameters are simultaneously correlated (such as pH, electrical conductivity, EE%, turbidity, and organoleptic properties). Furthermore, the biodegradability of these carriers in colloidal systems is a natural process, so the development of preservative-containing formulations allows the delaying of the particle destabilization.

The developed formulation containing different preservatives was fully characterized. Encapsulation Efficiency (EE%) was evaluated using Ultraviolet–Visible (UV–VIS) spectroscopy. Nanoparticle Tracking Analysis (NTA) and zeta potential were used for particle size distribution and surface charge measurements, respectively. Atomic Force Microscopy (AFM) allowed the evaluation of the particle’s morphology and surface roughness parameters. The stability evaluation of the developed systems followed the Cosmetic Products Stability Guide [29], based on *(i)* preliminary stability, *(ii)* stability under constant handling at (25 ± 2) °C, and *(iii)* shelf-life tests at (25 ± 2) °C and (35 ± 2) °C. The most stable system was analyzed using Laser Scanning Confocal Microscopy (LSCM). Fluorescence measurements and Attenuated Total Reflectance–Fourier-transform Infrared Spectroscopy (ATR–FTIR) were applied specifically to analyze the complexation of the essential oil and carrier. Finally, the controlled release [31,32] of essential oil was evaluated under different pHs (4, 7, and 10).

The use of nanotechnology allows the development of alternative materials able to enhance the stability and activity of natural controlling agents [33,34]. For this reason, the goal of this paper was to propose a new colloidal system with high EE% and stability as a function of time, so that the delivery of the active compounds occurs under controlled release over time and at desired targets, based on the effectiveness of *P. nigrum* essential oil. Besides the evaluation of the system’s stability as a function of different preservatives, a pulsatile mechanism of release was obtained herein and systematically correlated to the chemical interaction between the essential oil and particle carriers.

## 2. Materials and Methods

### 2.1. Essential Oil Extraction and Characterization

*Piper nigrum* seeds (SISGEN Authorization code A26CD5E) were purchased at the Adolpho Lisboa market in Manaus/AM. For essential oil extraction, 150 g of dried seeds were subjected to hydrodistillation using a modified Clevenger-type apparatus for 4 h at 100 °C and stored at −20 °C. Essential oil yield was obtained by taking the ratio between the extracted oil volume and the seed mass. Relative density of the essential oil was estimated at 20 °C and was converted by the water table density [35]. The refraction index of the raw essential oil was estimated at 20 °C using an Atago Master-T series refractometer (Ribeirão Preto, Brazil).

GC-FID analysis was carried out using a Shimadzu™ GC2010-FID instrument (Kyoto, Japan) equipped with a Flame Ionization Detector (FID) and a DB-5 (0.25 mm × 30 m, 0.25 μm coating thickness) fused silica capillary column. The operating conditions were as follows: injector and detector temperatures were 250 °C and 290 °C, respectively; helium was used as carrier gas at a flow rate of 1.0 mL·min^−1^; the column was heated from 60 °C to 250 °C with a rate of 3 °C·min^−1^. The split ratio was 1:10. GC–MS analysis was performed using a Shimadzu™ GCMS-QP2010 instrument (Kyoto, Japan) with the same column used in the CG-FID analysis and the same operating conditions. The MS profile was obtained at 70 eV with an acquisition mass range of 32–420 Da. Identification of the isolated compounds was established from their GC retention index, using a C_7_–C_30_ *n*-alkanes homologous series, whose arithmetic index (AI) was calculated using the Dool–Kratz equation [36]. The identification of the essential oil compounds was successfully confirmed [37,38,39,40].

### 2.2. Nanoparticle Development and Essential Oil Encapsulation

Colloidal system development was based on previous reports, with some modifications [28]. Gelatin was heated to 50 °C in distilled water under constant stirring. Then, Tween 80 (0.3 g) was solubilized when the gelatin solution reached 40 °C (solution I). Solution II was prepared using PCL (0.05 g), span 60 (0.02 g), and caprylic/capric triglyceride acid (0.1 g) solubilized in dichloromethane (5 mL). *P. nigrum* (SISGEN authorization code A26CD5E) essential oil (500 µg·mL^−1^) was added to solution II. Finally, solution II was slowly added to solution I, by applying an ultra-disperser (Mylabor, São Paulo, Brazil) (10,000 rpm). Transglutaminase (0.09 g) was added to the final colloidal systems and maintained under constant stirring (25 °C) for solvent evaporation. The same methodology described above was performed without *P. nigrum* essential oil (in solution II), to obtain unloaded particles. The preservatives *(i)* phenoxyethanol/methylisothiazolinone (PNE), *(ii)* methylisothiazolinone (PMI), *(iii)* sodium benzoate (PBS), *(iv)* thymol (PTI), or *(v)* ethylenediaminetetraacetic acid (PED) were added to the system containing encapsulated essential oil. An encapsulated system containing no preservative (PSC) was also prepared.

EE% was evaluated using UV–VIS spectroscopy [41]. A known concentration of essential oil in ethanol was scanned in the range of 190–400 nm on a Epoch2 Microplate Reader Biotek (Agilent, Santa Clara, CA, USA). A sharp peak at 278 nm was noticed. From the calibration curve, the unknown concentration of essential oil was obtained by knowing the absorbance values. Particles were separated by centrifugation (Daiki Sciences, Seoul City, Korea) (20,000 rpm). The supernatant absorbance was used to determine the percentage of free essential oil, using the formula: (EE%) = (amount of encapsulated essential oil/total amount of essential oil used in the formulation) × 100. Experiments were carried out in triplicate.

### 2.3. Zeta Potential

Zeta potential values (in mV) were measured on a Zetasizer Nano ZS90 device (Malvern Instruments, Malvern, UK) from pH = 2 to 12 at 25 °C in triplicate.

### 2.4. Atomic Force Microscopy (AFM)

Colloidal systems containing unloaded or loaded particles were dripped on a glass slide and allowed to dry. Measurements were performed at (296 ± 1) K and (40 ± 1) % R.H. on a Innova (Bruker^®^, Billerica, MA, USA) equipment operated in tapping mode and equipped with a silicon tip and Al coated cantilever with a spring constant of 42 N/m (Tap190AL-G) (Budget Sensors^TM^, Sofia, Bulgaria). Scans were performed using (10 × 10) µm^2^ with (256 × 256) pixels at a scan rate of 1Hz.

Images were analyzed using the software WSxM 5.0 [42], providing the mean square roughness (R_q_) and the arithmetic mean roughness (R_a_) of the surfaces. These parameters can be calculated using the following equations [43]:

(1) $$R_{q}=\sqrt{\frac{1}{N_{x}N_{y}}\overset{N_{x}}{\underset{i=1}{\sum }}\overset{N_{y}}{\underset{j=1}{\sum }}{\left(z(i,j)-\frac{1}{N_{x}N_{y}}\overset{N_{x}}{\underset{i=1}{\sum }}\overset{N_{y}}{\underset{j=1}{\sum }}z_{ij}\right)}^{3}}$$

(2) $$R_{a}=\frac{1}{N_{x}N_{y}}\overset{N_{x}}{\underset{i=1}{\sum }}\overset{N_{y}}{\underset{j=1}{\sum }}\left|Z(i,j)-\frac{1}{N_{x}N_{y}}\overset{N_{x}}{\underset{i=1}{\sum }}\overset{N_{y}}{\underset{j=1}{\sum }}Z_{ij}\right|$$

where *N_x_* and *N_y_* represents the number of points on the *x* and *y* axes, respectively. They represent, respectively, the standard deviation of the surface height distribution and the absolute mean deviation of irregularities in the roughness of the surfaces midline. The software ImageJ [44] was used for particle size distribution.

### 2.5. Nanoparticle Tracking Analysis (NTA)

Size characterization was performed on a NanoSight NS300 device (Malvern Instruments, Malvern, UK), equipped with a green laser type and a SCMOS camera. Data collection and analysis was performed using the software NTA 3.0 (Malvern Panalytical, Malvern, UK). Samples were diluted in MilliQ water (1:100 *v*/*v*). Measurements were performed in triplicate at 25 °C. The evaluation of the particle size distribution (PSD) was performed with the parameters Mean, Mode, SD, D_10_, D_50_ (Median), and D_90_, which indicate, respectively, the average, most frequent particle class size, standard deviation, and the 10%, 50%, and 90% percentiles of the analyzed particles. Specifically, D_10_, D_50_, and D_90_ indicate, respectively, the size below which 10%, 50%, and 90% of the total number of particles was included; mean and mode point to the average particle size and the most represented size value; while SD is the standard deviation of the distribution.

### 2.6. Stability Evaluation

The stability evaluation of the developed systems were based on the Cosmetic Products Stability Guide [29], with some modifications. All systems were previously submitted to a centrifugation test at 3000 rpm for 30 min. Then, the stability tests were performed as follows:

(i) Preliminary stability: all systems were subjected to (5 ± 2) °C for the first screening.
(ii) Stability under constant handling at (25 ± 2) °C: Colloidal systems containing preservatives (PNE, PMI, PTI, PED, and NBS) were stored in transparent vials and maintained in a bio-oxygen demand (BOD) incubator at (25 ± 2) °C. All systems were subjected to constant handling (vials were opened and exposed to the laboratory environmental conditions such as air contact, light, and temperature variation) at pre-established time intervals (1–3 days) until reaching an EE% equal to or less than 70%, or presenting alterations (slightly (SA) or intensely Altered (IA)) of their organoleptic properties (color and odor). Physical parameters (pH, electrical conductivity, EE% and turbidity) were measured in all vials on opening. All measurements were performed in triplicate.
(iii) Shelf-life test: The systems selected in the stability test under constant handling at (25 ± 2) °C were submitted to shelf-life tests at (25 ± 2) °C and (35 ± 2) °C. Systems were stored in sealed vials until reaching an EE% equal to or less than 70%, or presenting alterations ((SA) or (IA)) in organoleptic properties (color and odor). Vials were opened every 30 days. Physical parameters (pH, electrical conductivity, EE%, and turbidity) were measured in all vials on opening. All measurements were performed in triplicate.

### 2.7. Laser Scanning Confocal Microscopy (LSCM) and Fluorescence Measurements

Images were taken on a Carl Zeiss microscope (inverted model LSM 780) (ZEISS Research Microscopy Solutions, Jena, Germany), with Ti: Sapphire LASER, a 40× objective lens, 1.2 NA, and a 0.28-mm work distance. Systems containing unloaded and loaded particles (PNE) were centrifuged. Part of the supernatant was discarded for visualization of the largest particles. Then, a few drops were placed on a microscopy glass slide and analyzed directly in a confocal microscopy. Measurements of fluorescence were carried out with a 63× objective and SPAD (single photon avalanche diode) detector with a temporal resolution of 70 ps. A Coherent Chameleon tunable 690–1100 nm laser was used as the excitation source. Measurements were taken at 800 nm.

### 2.8. Attenuated Total Reflectance–Fourier-Transform Infrared Spectroscopy (ATR–FTIR)

ATR-FTIR analysis was performed on a Cary 630 FTIR spectrophotometer (Agilent, Santa Clara, CA, USA), in the range 2000 cm^−1^ to 650 cm^−1^.

### 2.9. Controlled Release

A colloidal system containing the loaded particles (15 mL) was inserted into a dialysis tubing cellulose membrane and suspended in water (85 mL; 25 °C) at pH = 4, 7 or 10. The system was maintained under continuous magnetic stirring (100 rpm). A 3 mL aliquot was withdrawn from the flask at regular time intervals (up to 220 h). Absorbance was measured at 278 nm on a Epoch2 Microplate Reader Biotek. The amount of released essential oil was calculated from a standard curve. The cumulative release (%) of essential oil was obtained from the following equation: [Cumulative release (%) = (amount of essential oil released after time *t*/total amount of encapsulated essential oil) × 100] [45].

## 3. Results and Discussion

### 3.1. Essential Oil Characterization

The optimal extraction time of essential oil from *P. nigrum* seeds was found to be 3 h, and this influenced the essential oil yield. The highest yield (approximately 2.6%) was observed after 3 h and was maintained until 4 h of extraction. The RI of the extracted essential oil was estimated as (1.48 ± 0.01). The density was calculated at around (0.78 ± 0.01) g.cm^−3^. Similar values of RI and density were reported previously [46].

The chemical compounds of the essential oil were identified by GC–FID/GC–MS, and this allowed the identification of 29 constituents, representing 95.32% of the total compounds. The major component was *β*-caryophyllene (34.87%), followed by sabinene (14.96%) and sylvestrene (14.17%). Other identified terpenes were found below 5.28%. A similar composition was reported previously [47]. However, some reports identified *β*-caryophyllene as ranging from 25% to 80% of the total composition [46,48,49,50,51,52,53], due to the different climate, cultivation/soil, and extraction methods, as well as nutrient deficiencies and the presence and/or absence of pathogenic microorganisms. On the other hand, other reports did not identify *β*-caryophyllene as a major compound of this essential oil [54].

*β*-caryophyllene [52,55] is a bicyclic sesquiterpene found in other aromatic species [46,56,57,58]. Anti-inflammatory, antibiotic, antioxidant, anticarcinogenic, and anesthetic properties have been attributed to this compound [59]. Recently, promising hepatoprotective and antifungal activities were reported [60]. However, some reports indicated that chemical synergism is responsible for the biological activity of the *P. nigrum* essential oil [22,52,61,62].

### 3.2. Particle Size Evaluation

**Figure 1:**
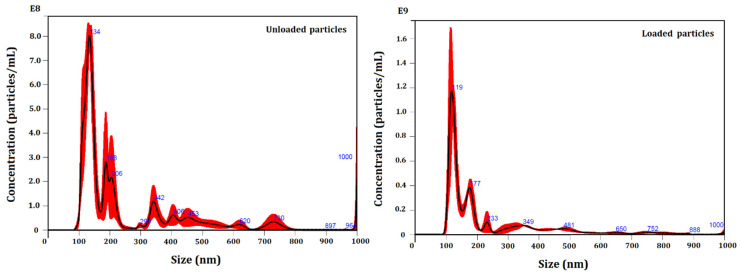
NTA particle size distribution analysis of unloaded and loaded systems. Representative histograms of the average size distribution (black line) from three measurements of a single sample. Red areas specify the standard deviation (SD) between measurements, and blue numbers indicate the maxima of individual peaks.

Unloaded and loaded particles were characterized for their number and size distribution by NTA (Figure 1). Table 1 shows the average particle size of the unloaded and loaded systems.

The developed colloidal systems were compared in terms of both size and concentration (particles/mL) as a function of the encapsulated essential oil. A slight change of size and particle concentration was observed, as registered by all the size descriptors. This reduced size diameter may be related to interactions between the essential oil and carriers, as will be discussed in the following sections. The systems presented a polydisperse particle size distribution, ranging from (114 ± 3) nm to (519 ± 13) nm. Moreover, 90% of the particle population constituting the unloaded and loaded systems presented size up to (519 ± 13) nm and (456 ± 11) nm, respectively.

The mode parameter shows the particle size (or size range) most commonly found in the population distribution, and this is helpful to describe the midpoint for nonsymmetric distributions [63]. The value that best represented the loaded system was (122 ± 4) nm. Our results showed that the particle size distribution profile was not significantly influenced after the encapsulation of the essential oil, but the particle size presented a slight reduction, as registered by all the size descriptors.

### 3.3. AFM Analysis

**Figure 2:**
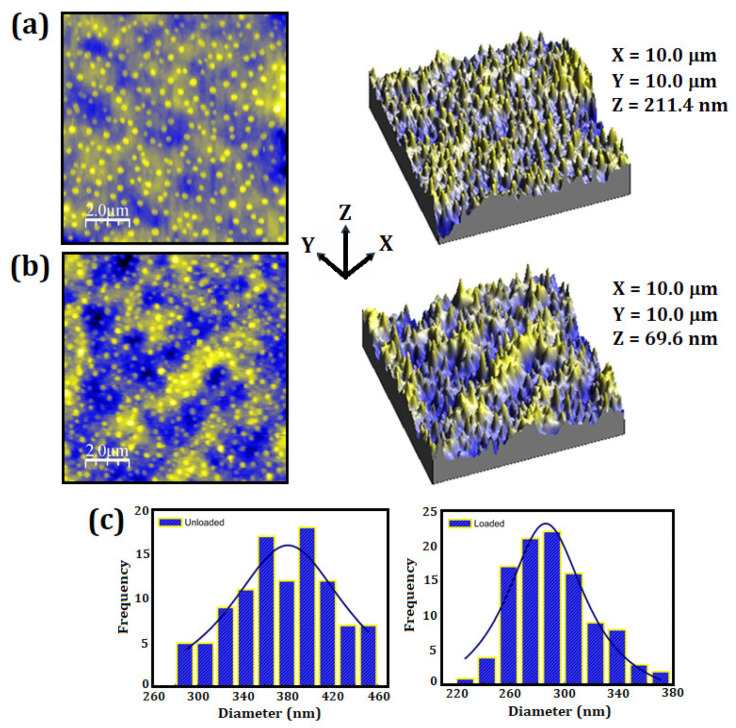
Two-dimensional and three-dimensional AFM micrographs: (**a**) unloaded particles, (**b**) loaded particles, (**c**) size distribution of unloaded and loaded particles.

Figure 2 shows the micrographs of the unloaded and loaded particles, which presented an almost spherical morphology. The average size of the unloaded particles (Figure 2a) was found at around (366 ± 43) nm and a PDI of (0.17 ± 0.03). The loaded particles (Figure 2b) presented an average size of (284 ± 30) nm and PDI of (0.23 ± 0.03). Spherical morphology, as well as regular distribution of peaks and valleys along the particle surface (Figure 2c), are significantly in a controlled release formulation, due to the required uniform adhesion on another surface [64]. The AFM results presented differences of the average size distribution for unloaded and loaded particles. However, both techniques pointed to the decreased diameter of the loaded particles. Moreover, it is important to consider the sensitivity of NTA and AFM equipment [65].

The surface roughness of the unloaded and loaded systems was evaluated through four different topography images, obtained in different regions of the surface of each sample. Figure 2a,b shows representative images of the surface of the unloaded and loaded particles, respectively. The arithmetic mean surface roughness of particles (Ra) is defined as the mean absolute deviation of irregularities in midline roughness along a surface length [66]. The mean square roughness (Rq) is the standard deviation of the surface height distribution and describes the surface roughness. The Ra and Rq values obtained for the unloaded particles were (9 ± 1) and (11 ± 2), respectively. Considering the loaded particles, Ra = (15 ± 1) and Rq = (20 ± 2) were obtained. These results revealed the influence of the essential oil on the surface roughness of particles after the encapsulation process [67]. Our results suggested that the loaded particles presented better conditions for application as a releasing product, due to its higher roughness and microtexture, favoring an efficient adhesion on another surface [68].

### 3.4. Zeta Potential (ζ)

The zeta potential is an important physicochemical parameter, related to the stability of colloidal systems [69]. The variation of the zeta potential as a function of pH for the unloaded and loaded particles was evaluated (data not shown). Higher values (in module) of zeta potential are related to significant repulsion and reduction of aggregation/agglomeration [70]. There was a tendency for both systems to maintain their isoelectric points (ζ ~ 0) from pH = 2 to pH = 4. The isoelectric point was verified as close to pH = 4 and was related mainly to the type B gelatin carrier. It is known that two types of gelatin (A or B) can be produced, depending on the method in which collagen is pretreated [71]. The isoelectric point of gelatin can be modified during its extraction from collagen, to yield either a negatively-charged acidic gelatin, or a positively-charged basic one [72]. However, between pH = 5 and pH = 8, both systems presented an increased surface charge, reaching the highest value (in module) at pH = 8: ζ = (–35 ± 2) mV and ζ = (–45 ± 3) mV, respectively, for the unloaded and loaded systems.

The analysis of the zeta potential is also an indicator of the stability of colloidal systems. Electrostatic stability occurs when there is a repulsion between the particles due to the high surface charge [73]. Loaded particles presented a higher surface charge (in module), probably due to the presence of essential oil and due to rearrangements among their constituents, resulting in improved stabilization [74]. Similar values of zeta potential were found previously [75]. The authors prepared and characterized micro and nanoemulsions containing essential oil from *P. nigrum* by ultrasound and high-pressure homogenization using span 60 and tween 80. The zeta potential values were found to be between (–48 ± 1) mV and (–51 ± 2) mV.

### 3.5. Stability of Loaded Particles Containing Preservatives

The EE% value of the loaded particles was estimated as (98 ± 2) %. A high EE% has been reported for particles constituted of gelatin/PCL [28,74]. The encapsulation of bioactive compounds within the carrier particles is related to the chemical nature of the encapsulated bioactive (including its molecular weight, chemical functionality, polarity, and volatility), in addition to the properties of the carrier and the encapsulation methodology [76].

Various essential oils are encapsulated to preserve and improve their bioactivity, and the great challenge is to create chemical mechanisms such that the EE% is maintained for as long as possible. Few reports have evaluated the variation of the EE% as a function of time [10,77]. The addition of preservatives in colloidal systems represents an alternative to enhancing the formulation stability. The industrial applicability of colloidal systems based on polymeric or biodegradable particles is limited, due to their reduced physicochemical stability over long periods of storage. The main limitations are the particle aggregation, chemical stability of the carrier, and the undesirable exposure of the bioactive substance to the solution medium [69,78,79]. Furthermore, it is important to emphasize that biodegradable formulations are susceptible to microbial proliferation [10].

#### 3.5.1. Preliminary Stability

Preliminary stability is known as a screening test for accelerated or short-term stability. This test employs extreme temperature conditions, in order to accelerate possible reactions between the components of the formulation. Therefore, this test is not intended to estimate the shelf-life, but to assist in screening for the most stable system [29].

The systems containing encapsulated essential oil and preservatives (PNE, PMI, PBS, PTI, and PED) were stored at (5 ± 2) °C. Viscosity and phase separation were observed after 24 h, as well as flocculation and coalescence. All systems showed larger agglomerates separated from the rest of the emulsion, as reported previously [80]. The developed systems were not suitable for storage at low temperatures, as expected, due to the presence of the gelatin carrier.

Then, all systems were maintained in a BOD incubator at (35 ± 2) °C for a preliminary evaluation of their stability (these systems were not those subjected to (5 ± 2) °C). The results are shown in Table 2. The systems were opened after 15 days, and the parameters EE%, electrical conductivity, turbidity, pH, and organoleptic properties (color and odor) were evaluated.

After 15 days of evaluation at (35 ± 2) °C, all systems maintained their EE% values above (70 ± 2)%, which is considered representative [81].

The pH, electrical conductivity, and turbidity parameters have been generally associated with the decrease of EE%, due to the exposure of essential oil (previously encapsulated) to the solution medium. The PNE, PMI, and PTI systems maintained their EE = (98 ± 2)% after 15 days. Considering the other systems, PBS and PSC reached the lowest EE% values, around (76 ± 2)%. Preliminarily, we suggested that the preservative sodium benzoate was the least efficient, since the EE% of the PBS system was similar to that of the encapsulated system with no added preservative (PSC).

Monitoring pH also represents an important tool to evaluate the stability of colloidal systems [82]. Changes can be associated with the decrease of EE% and, consequently, with exposure of essential oil to the solution medium. After 15 days, the lowest pH values were observed in systems presenting lower EE%, such as PBS and PSC. Although the PNE and PMI systems maintained their EE%, a marginal decrease of their pH values was observed, probably due to the natural interactions between the constituents of the formulation [83].

The electrical conductivity increased in all systems after 15 days. The highest value was observed in the PSC system (from (628 ± 5) µS·cm^−1^ to (5000 ± 10) µS·cm^−1^). Changes of electrical conductivity were also related to the essential oil exposure to the solution medium. Essential oil constituents can be converted into other compounds by oxidation, isomerization, cyclization, or dehydrogenation [84]. According to GC/FID and GC/MS data, the essential oil from *P. nigrum* is rich in compounds containing electronegative groups that can contribute to the electrical conductivity [84]. On the other hand, the exposure of the essential oil to the medium is a consequence of the particle destabilization, resulting in chemical reactions that may release free electrons [30].

Corroborating the indications of EE% and pH, the analysis of turbidity also showed changes over time, especially in those systems presenting a more significant decrease of EE%. This result may be related to the particle destabilization and successive release of essential oil into the medium, besides particle fragments [82]. The PNE and PMI systems showed the smallest turbidity variation, increasing from (40 ± 1) NTU to (45 ± 2) NTU in the PNE system, and remaining at (40 ± 1) NTU in the PMI system.

The organoleptic properties (color and odor) remained unchanged only in the PNE and PMI systems. Modification of organoleptic properties is usually associated with the proliferation of microorganisms [29], suggesting a better action of the phenoxyethanol/methylisothiazolinone and methylisothiazolinone preservatives.

Higher temperature of storage can also lead to chemical reactions responsible for changing the evaluated parameters influencing the reaction kinetic and material degradation. Gelatin can undergo a gelatinization process when it is maintained above room temperature for a long time [85]. However, this phenomenon was not observed, since some systems (such as PNE and PMI) maintained their high EE% over the evaluation period. Furthermore, nanometric gelatin may exhibit a different behavior when compared to that of the bulk powder form [86].

In general, changes of pH, electrical conductivity, and turbidity were basically related to the exposure of the essential oil to the solution medium. Although the PBS, PED, PTI, and PSC systems presented slight variations of color and odor, we still chose to keep them in the subsequent evaluation at (25 ± 2) °C, in order to verify the influence of temperature on their stability. However, the preliminary stability results pointed to PNE and PMI as the most stable systems, which presented marginal variations of EE%, electrical conductivity, pH, turbidity, and organoleptic properties as a function of time.

#### 3.5.2. Stability under Constant Handling

This test was performed to estimate the shelf-life of all systems when they were subjected to conventional handling conditions, such as exposure to light, air contact, as well as temperature changes (from (25 ± 2) °C to (17 ± 2) °C, local laboratory temperature).

- EE% and Electrical Conductivity

**Figure 3:**
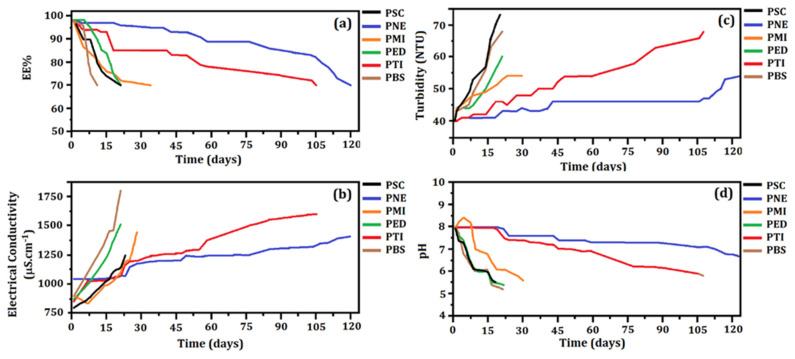
(**a**) Encapsulation Efficiency (EE%), (**b**) electrical conductivity, (**c**) turbidity and (**d**) pH of the loaded systems containing preservatives as a function of time (days).

Figure 3a shows the evolution of EE% as a function of time for all systems, which presented an EE = (98 ± 2) % (except for the unloaded particles system, PSC). The achieved high EE% showed the success of the developed loaded particles. However, the evaluation of the EE% is not sufficient to guarantee the stability of the formulation for a long time period.

The first system to reach EE = 70% under constant handling was the PBS after 13 days. This system was less stable than the PSC. The sodium benzoate preservative may have contributed to the system destabilization. The conversion into benzoic acid reduced the pH of the formulation, leading to its isoelectric point [87].

The PSC and PED systems reached EE = 70% after 20 days. The PMI system showed a similar stability to that of the PSC, PBS, and PED systems, reaching the considered limit of EE% after 34 days. Therefore, the preservatives sodium benzoate and EDTA were not efficient in maintaining stability, since the EE% of these systems reached 70% in a time equal to or less than the PSC system. The lower efficiency of these preservatives could also be verified by evaluating their organoleptic properties, which were intensively altered (IA).

The systems that clearly showed greater stability at (25 ± 2) °C as a function of time were PTI and PNE. The PTI system reached EE = 70% after 105 days. Thymol has been used in antiseptic formulations with antifungal and antibacterial properties, and can also be found as the major constituent of thyme (*Thymus vulgaris*) essential oil [88].

The PNE system presented the highest stability, reaching EE = (70 ± 2)% after 120 days. The combination of different preservatives (in this case phenoxyethanol and methylisothiazolinone) has shown effectiveness for a wide spectrum of fungi and bacteria [89,90]. Considering that the PSC system reached EE = 70% after 20 days and the PNE system reached the same EE% after 120 days, we can suggest that the NE preservative increased the stability of the PNE system in 100 days.

The results of the stability evaluation as a function of electrical conductivity are shown in Figure 3b. The PSC system showed a variation of electrical conductivity from (800 ± 4) µS·cm^−1^ to (1250 ± 4) µS·cm^−1^ after approximately 21 days. An increase of electrical conductivity in all systems was observed over time, after the addition of preservatives. The values ranged from (875 ± 5) µS·cm^−1^ to (1509 ± 5) µS·cm^−1^ in the PED system; from (900 ± 4) µS·cm^−1^ to (1446 ± 4) µS·cm^−1^ in the PMI system, and from (900 ± 5) µS·cm^−1^ to (1800 ± 5) µS·cm^−1^ in the PBS formulation. These systems presented significant variations of electrical conductivity and reached EE = 70% within 30 days. Thus, the increased electrical conductivity may be related to the exposure of essential oil into the solution medium [10,84], probably due to the particle disruption [30].

The PTI system presented electrical conductivity between (856 ± 5) µS·cm^−1^ and (1600 ± 5) µS·cm^−1^ after 105 days, and the PNE system showed an increase of electrical conductivity from (1043 ± 5) µS·cm^−1^ to (1410 ± 5) µS·cm^−1^ after 120 days. These values corroborated the results obtained from EE%. Changes of electrical conductivity of the dispersed systems may be related to the particle disruption, while the decrease of electrical conductivity was related to the particles aggregation [29]. Thus, we can suggest that the electrical conductivity of these systems increased due to the exposure of the essential oil to the medium (as the EE% decreased by the disruption process). This result was also associated with the observed turbidity variation.

- Turbidity and pH

Figure 3c shows the evolution of turbidity as a function of time for all systems. At *t* = 0, all systems presented a turbidity around (40 ± 1) NTU. This fact shows that the addition of preservatives did not influence the initial turbidity values. However, as in the evaluation of EE% and electrical conductivity, the greatest turbidity variations were observed in the PSC, PMI, PBS, and PED systems.

The turbidity reached (75 ± 3) NTU in the PSC system, (68 ± 2) NTU in PBS, (60 ± 2) NTU in PED, and (54 ± 4) NTU in the PMI within 30 days. This result may be related to the particle destabilization, since suspended fragments was visible in the medium.

The turbidity of the PTI system reached (68 ± 2) NTU after 105 days, while the PNE system reached (54 ± 4) NTU after 120 days. The turbidity variation for these two systems remained similar to the variation of the EE% and electrical conductivity: the turbidity values increased as the systems gradually destabilized, accompanied by a decrease of EE% and increase of electrical conductivity [29].

The pH variation as a function of time is shown in Figure 3d. According to the zeta potential analysis, all systems were initially adjusted to pH = 8. The PMI, PBS, PED, and PSC systems showed abrupt changes of pH after approximately 30 days, when these systems reached EE~70%. The pH of the PSC, PED, PBS, and PMI systems decreased to (5.40 ± 0.03), (5.40 ± 0.03), (5.20 ± 0.02), and (5.60 ± 0.03), respectively. The decrease of pH can possibly be explained by the exposure of essential oil to the medium (as a consequence of the particles disruption. This suggestion was based on the results of the EE%, electrical conductivity, turbidity, and AFM) [47]. As the pH of the essential oil from *P. nigrum* was approximately (5.40 ± 0.04), the systems became more acidic when the essential oil was exposed [10]. However, the decrease of pH over time in the colloidal systems may also be associated with the degradation of the materials contained in the formulation, inducing acidification [82]. This result may also indicate polymeric degradation due to the ionization of carboxylic groups and hydrolysis [83]. The possibility of gelatin degradation by reactions with free carboxylic groups can also be suggested [91].

The pH of the PTI system remained stable for 18 days at (8.00 ± 0.02), showing a slight decrease to (7.50 ± 0.04) and then remaining stable for approximately 31 days. Then, there was a reduction to pH = (5.80 ± 0.03) after 105 days. The PNE formulation presented the most regular variation of pH, which remained at (8.00 ± 0.02) for 21 days. After a slight decrease, it remained at (7.50 ± 0.04) for 89 days, and then the evaluation concluded at pH = (6.80 ± 0.01) after 120 days.

In all systems, the pH tended to reach the most acidic scale, and this fact may have contributed (in addition to the destabilization of the particles and exposure of the essential oil) to the tendency of the surface charges to reach the isoelectric point (pH = 4). The evaluation of stability under constant handling showed that, at (25 ± 2) °C, two preservatives contributed significantly to maintaining the stability: NE and thymol. Destabilization phenomena were also observed in these systems (PNE and PTI); however, this occurred slowly and gradually in all evaluated parameters. Understanding the behavior of these physical parameters could be useful for adjusting both the components and concentration of the formulation ingredients.

**Figure 4:**
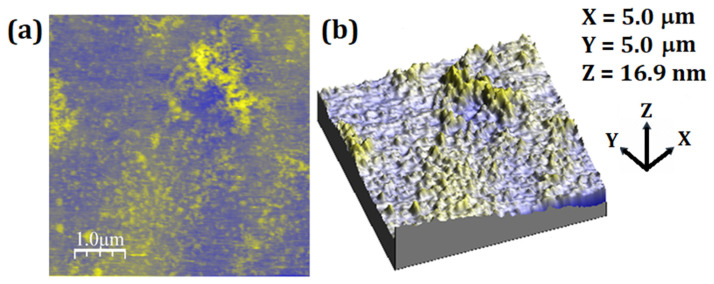
Two-dimensional and three-dimensional AFM micrographs: (**a**) PNE system destabilized, and (**b**) topographic map.

To visually verify the particles destabilization, the fully destabilized PNE system was subjected to AFM analysis. Figure 4 shows the 2D and 3D images of this system. The disappearance of the particles previously seen in the stabilized system is clear. Furthermore, it is possible to verify particle fragments resulting from the rupture.

- Organoleptic Properties

**Figure 5:**
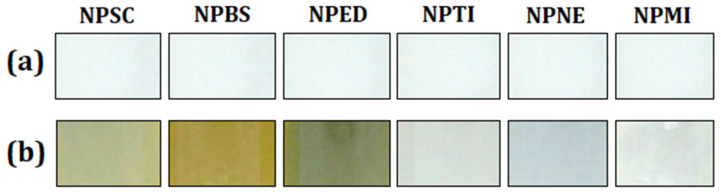
Color evaluation of the systems maintained under constant handling at (25 ± 2) °C: (**a**) control system, and (**b**) systems after evaluation.

The color evaluation of the systems maintained under constant handling at (25 ± 2) °C is shown in Figure 5. All systems were compared with the control formulation.

The PNE and PTI systems presented significant color changes. In addition, these systems showed no odor change after the evaluation time. On the other hand, the PBS, PED, and PSC systems showed intense changes of color and odor, becoming a yellowish color. The PMI system did not show an intense color change, but its odor was intensely altered. Changes of color are usually related to chemical changes of formulation and/or proliferation of microorganisms [29].

The PSC, PBS, PED, and PMI systems also presented sedimentation fragments [92], corroborating the particle rupture [80]. The odor of these systems remained intensively modified. Only the PNE and PTI systems did not show any color/odor changes or sedimentation fragments.

#### 3.5.3. Shelf-Life Evaluation at 25 °C and 35 °C

A shelf-life evaluation at 25 °C was performed only for the PNE and PTI systems. The PSC system was used as a control. The results are shown in Table 3.

The PNE system maintained stability for 210 days, considering no alteration (NA) of its organoleptic properties. The EE% decreased around by 14% ((from (98 ± 2) % to (84 ± 1) %), with a final pH of (7.40 ± 0.09), turbidity equal to (50 ± 2) NTU, and electrical conductivity of (1532 ± 4) µS·cm^−1^. Then, between 210 and 270 days, this system presented slightly altered organoleptic properties (SA), reaching EE = (70 ± 1)% after 270 days of evaluation.

The PTI system maintained stability for 120 days, considering no alteration (NA) of its organoleptic properties. The EE% decreased by around 18% ((from (98 ± 3) % to (80 ± 2) %), with a final pH of (7.00 ± 0.02), turbidity equal to (48 ± 1) NTU, and electrical conductivity of (1342 ± 5) µS·cm^−1^. Then, between 150 and 210 days, this system presented slightly altered organoleptic properties (SA), reaching EE = (70 ± 2)% after 210 days of evaluation.

The PSC system presented slightly altered organoleptic properties (SA) within 30 days.

The variation of pH, turbidity, and electrical conductivity of the PNE, PTI, and PSC systems followed the same criteria as described previously and was directly related to the decrease of EE% as a function of time, due to the gradual particle rupture. The results revealed that the PNE system was the most stable over 210 days of storage at 25 °C in a transparent vial (the stability reached 120 days in the case of evaluation under constant handling). The PTI system also showed a good shelf-life stability, reaching 120 days of storage at 25 °C in a transparent vial (the stability reached 105 days in the case of evaluation under constant handling).

These results suggested that the action and efficiency of the NE and thymol preservatives were significant in delaying the particles destabilization. The prolonged stability of the PNE system may have been due to the combined preservative substances (phenoxyethanol/2-Methyl-2H-isothiazolin-3-one). Phenoxyethanol is a water-soluble organic compound, and its association with 2-Methyl-2H-isothiazolin-3-one (a hydrophilic heterocyclic organic compound) may have potentiated its action [89,90,93,94]. On the other hand, the preservative thymol (also effective against several microorganisms) [95,96] is reported to present a low stability when exposed to the environment and low solubility in aqueous media, in addition to being a volatile substance [97,98].

Shelf-life stability at 35 °C was also evaluated for the PNE and PTI systems. The PSC system was used as a control. Results are shown in Table 4.

The PSC system reached EE = 70% within 30 days.

The PNE system remained stable for 90 days, considering no alteration (NA) of its organoleptic properties. The stability at 35 °C, therefore, was lower than that observed at 25 °C (210 days). After 90 days at 35 °C, the EE% of the PNE system showed a reduction of 25% ((from (98 ± 2) % to (73 ± 2) %), with a final pH of (7.00 ± 0.08), turbidity equal to (49 ± 2) NTU, and electrical conductivity of (1070 ± 3) µS·cm^−1^. The final values of pH, turbidity, and electrical conductivity were similar to those observed in the evaluation at 25 °C. Only the conductivity value observed at 35 °C ((1070 ± 3) µS·cm^−1^) was lower than that observed at 25 °C ((1532 ± 4) µS·cm^−1^) after 210 days, probably due to the influence of the higher temperature [99]. Between 90 and 120 days, this system presented slight changes of organoleptic properties (SA), reaching EE = (70 ± 2)% after 120 days.

The PTI system remained stable only up to 30 days. The EE% decreased by about 28% ((from (98 ± 3) % to (70 ± 4) %), with a final pH of (5.00 ± 0.04), turbidity equal to (70 ± 2) NTU, and electrical conductivity of (1621 ± 4) µS·cm^−1^. After 30 days, this system showed intensively altered organoleptic properties (IA). Therefore, the stability at 35 °C (between 0 and 30 days) was lower than that observed at 25 °C (120 days).

The influence of temperature during storage at 35 °C was significant for the system destabilization. Shelf-life reduction was observed in the PNE (from 210 days at 25 °C to 90 days at 35 °C) and PTI (from 120 days at 25 °C to 0 days at 35 °C) systems. The preservatives NE and thymol are different in terms of their volatility, solubility, and bioactivity as a function of temperature. At higher temperatures, thymol tends to be eliminated from the systems more easily, due to its high volatility. However, all vials remained sealed during the shelf-life stability evaluation, suggesting that thymol may have been affected by temperature [98]. In the PNE system, the preservative was not significantly affected by the increase of temperature [90,93]. Although some reports state that gelatin can undergo destabilization when kept for a long time at high temperatures [100,101], this fact was not observed in the stability tests: at 25 °C and 35 °C, the PSC system was destabilized at the first measurement (*t* = 0).

For the reasons described above, we can conclude: *(i)* when stored at 25 °C both the PNE and PTI systems could be opened and applied within 7 and 4 months, respectively. This period showed that both systems were able to keep at least 70% of the essential oil encapsulated and maintained their organoleptic properties; and *(ii)* when stored at 35 °C, only the PNE system could be opened and used (within 3 months), since the PTI system did not show stability at this temperature. This period showed that the PNE system kept at least 70% of its essential oil encapsulated, besides maintaining its organoleptic properties.

### 3.6. Laser Scanning Confocal Microscopy (LSCM) and Fluorescence Measurements

**Figure 6:**
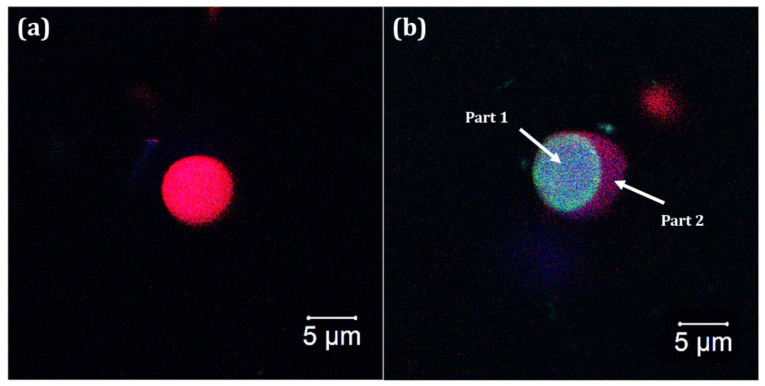
Confocal microscopy images of the particles from the (**a**) unloaded system and (**b**) loaded PNE system.

Figure 6 shows particle images from the unloaded and loaded PNE systems. Larger particles (µm) were selected to facilitate their visualization, as well as the essential oil in the loaded particles. According to the NTA measurements, 10% of the loaded particles were larger than (456 ± 11)]. The essential oil was clearly non-homogeneously located within the loaded particles, characterizing them as loaded capsules. Moreover, an absence of essential oil was observed in the unloaded system, as expected.

**Figure 7:**
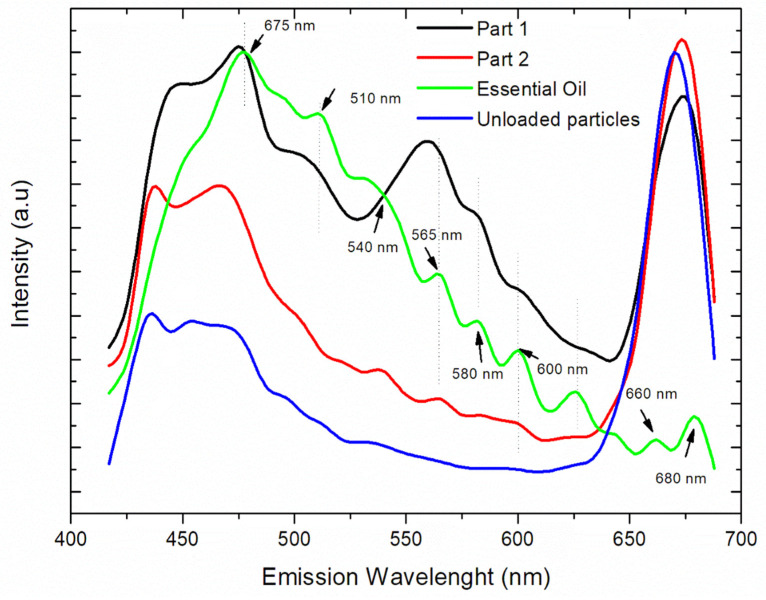
Fluorescence measurements of the loaded particle (part 1 and 2), essential oil in natura and unloaded particle.

Fluorescence measurements were also performed on the unloaded particles, as well as on two different parts of the loaded system, as highlighted in Figure 6b. Emission spectra are presented in Figure 7 and show that the fluorescence intensity was dependent on the essential oil content.

Distinct behaviors were observed in the loaded particles. The essential oil mainly presented emission peaks at 675 nm, 510 nm, 540 nm, 565 nm, 580 nm, 600 nm, 660 nm, and 680 nm. These peaks were observed in the emission spectrum of the PNE system (Part 2), confirming the presence of some amount of essential oil in this region. On the other hand, the emission peaks of the essential oil were not observed in the unloaded system, as expected. In this system, well defined and more intense emission peaks, mainly from the carriers (such as gelatin and PCL), were observed at 435 nm and 670 nm.

The intensity of the emission peaks of the essential oil (from 540–600 nm) was considerably enhanced in the emission spectrum of the PNE system (Part 1). This result suggested a more localized amount of essential oil within the particle, probably due to a complexation between the carriers and essential oil. It is important to stress that the localization of the essential oil within the loaded particles was observed in all selected particles during the analysis with a confocal microscope.

Fluorescence measurements have been widely used for the evaluation of interactions in the complexation of materials. The enhancement of the fluorescence intensity of *Salvia sclarea* L. essential oil (SEO), due to its complexation with *β*-cyclodextrin (*β*-CD), were investigated by spectrofluorimetry [102]. The introduction of *β*-CD to an aqueous solution of SEO resulted in dramatically enhanced fluorescence signals from the essential oil, due to complex formation. It has been generally accepted that one of the main driving forces for complexation are hydrogen binding and van der Waals force interactions between the host and guest molecules.

### 3.7. ATR-FTIR Analysis

**Figure 8:**
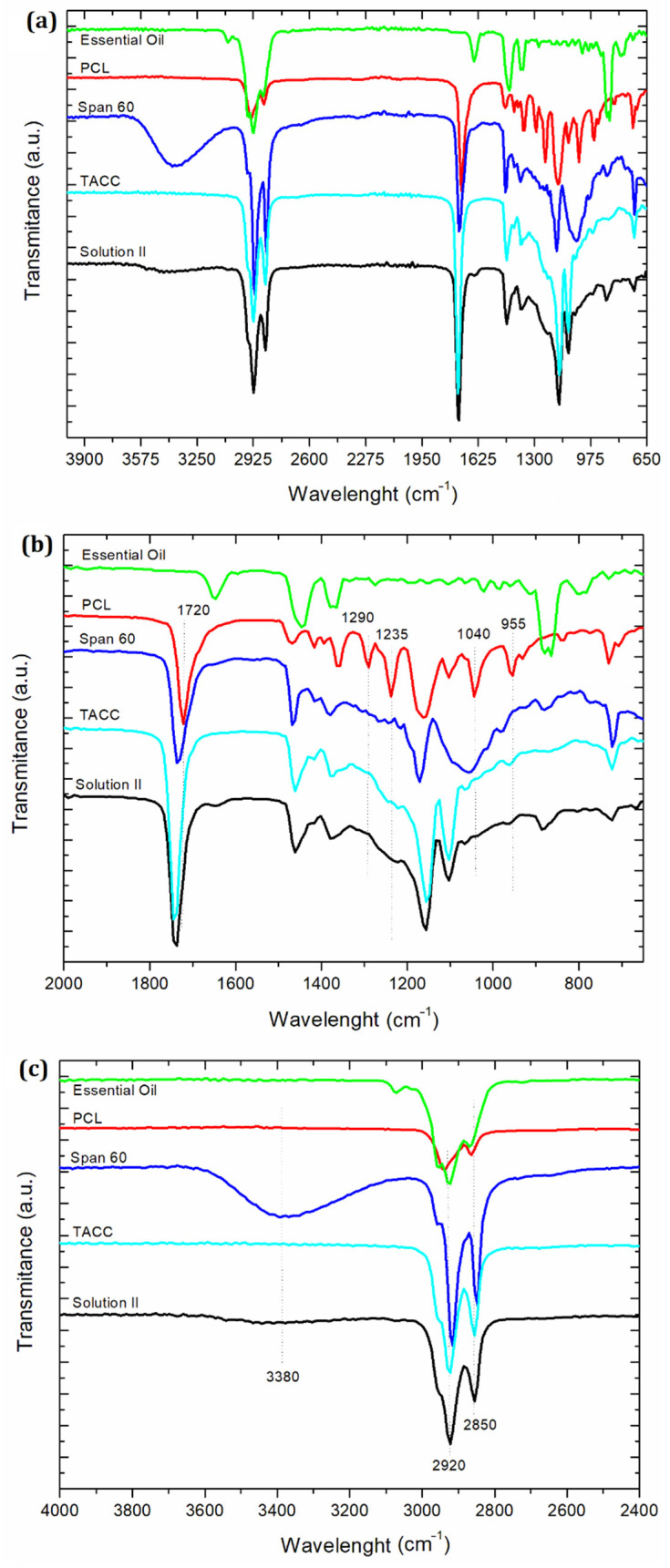
ATR-FTIR spectra of the *in natura* essential oil, PCL, span 60, TACC, and solution II (**a**) from 4000–650 cm^−1^, (**b**) from 2000–650 cm^−1^ and (**c**) from 4000–2400 cm^−1^.

In order to confirm the complexation of PCL with *P. nigrum* essential oil, solution II (prepared using PCL, span 60, TACC, and essential oil solubilized in dichloromethane) was subjected to ATR-FTIR analysis. Figure 8a shows the spectra of solution II and its individual constituents. With respect to PCL alone, we can easily identify in Figure 8b strong well defined bands, such as the carbonyl stretching (–C=O) at 1720 cm^−1^ [103]. C–O stretching in the crystalline phase was observed at 1290 cm^−1^. The peak at 1240 cm^−1^ was assigned to the asymmetric C–O–C stretching [104], as well as the C–O vibrations at 1047 cm^−1^ [105]. Stretching of the oxime bond at 955 cm^−1^ was also detected [106].

The disappearance and/or low intensity of peaks of the solution II observed mainly at 1290 cm^−1^, 1235 cm^−1^, 1040 cm^−1^, and 955 cm^−1^ were clearly attributed to the reduced availability of C–O of PCL upon complexation with essential oil. This result suggested the dispersion of some amount of essential oil in a PLC matrix, and it is also supported by the shift of the –C=O vibration from 1720 cm^−1^ (PCL) to 1740 cm^−1^ (solution II). In addition, the peaks of the span 60 and TACC spectra remained at the same positions, and the most intense peaks observed in the essential oil spectrum (1649 cm^−1^, 1445 cm^−1^, 1370 cm^−1^, 885 cm^−1^, and 867 cm^−1^) presented a reduced intensity in the solution II spectrum.

Figure 8c shows no changes in the peak formation/localization in the region around 3380 cm^−1^, nor in the asymmetric and symmetric CH_2_ stretching, respectively, at 2850 cm^−1^ and 2920 cm^−1^. For this reason, we suggested there was no chemical interaction by hydrogen bonding, but rather by complexation by van der Waals force interactions between the essential oil and the carboxyl groups of PCL. Part of the encapsulated essential oil was complexed with the PCL matrix and located within an internal region of the particle, while part of the non-complexed essential oil remained in another region. The total complexation of the essential oil was not observed, due to its hydrophobic behavior and poor solubility in water (justifying the use of span 60 as a lipophilic emulsifier), resulting in isolated droplets that were prevented from complexing. The ATR-FTIR results confirmed this information, corroborating the results from the confocal microscopy images, as well as the fluorescence spectra of the loaded particles. This result may also be related to the reduced size of the particles observed from the NTA results.

Interactions between carriers and actives have been extensively reported [47,75,107,108,109,110]. Similar results of interaction with PLC were also observed previously [105]. Reports on electrospinning of collagen using acetic acid in the presence of PCL have been resulted in a nano-fabric networks. The authors confirmed by FTIR analysis the interaction between PCL and collagen through hydrogen bonding, also reducing the availability of C–O in the developed blend [105].

### 3.8. Controlled Release

**Figure 9:**
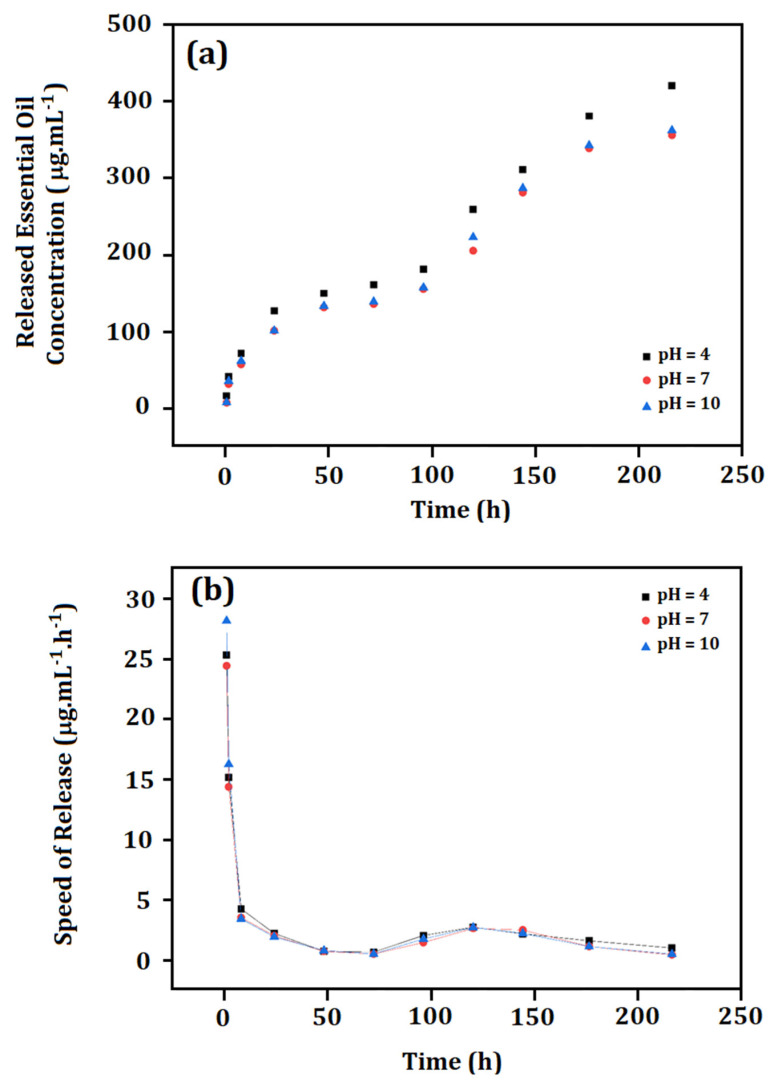
(**a**) Controlled release of essential oil at pH 4, 7, and 10, and (**b**) derived curve from the controlled release.

The controlled release curves of the essential oil at pH 4, 7, and 10, as well as the respective derivative curves are shown in Figure 9. The concentration of released essential oil was always slightly higher at pH = 4. Furthermore, the release of essential oil was significant for two different time intervals: *(i)* the speed of the released essential oil started at approximately 27 µg·mL^−1^/h and decreased until reach 3 µg·mL^−1^/h in the first 10 h. Thereafter, the releasing rate became slower up to 75 h (reaching 1 µg·mL^−1^/h). After 75 h, a second moment of controlled release was observed: *(ii)* the releasing rate increased again (between 75 h and 175 h, reaching a maximum peak of 3 µg·mL^−1^/h) and then was decreased. All curves presented this behavior.

The controlled release test was carried out for 225 h, and the total concentration of released essential oil was ((420 ± 2) µg·mL^−1^; 86%)) at pH = 4; ((355 ± 3) µg·mL^−1^; 72%) at pH = 7 and ((362 ± 2) µg·mL^−1^; 74% of released essential oil) at pH = 10. The highest amount of essential oil was released at pH = 4, and this can be explained by the greater tendency of destabilization in acidic pH, as shown in the zeta potential results.

The releasing curves (presenting different moments of higher concentrations of essential oil release; between 0 and 75 h, and between 75 h and 175 h) suggested a pulsed release mechanism [111,112]. This type of release is usually related to multilayer encapsulating systems, where the encapsulated active is probably located in different parts of the nanoparticles. In our case, this result was related to the complexation of some amount of essential oil in the PCL matrix.

Two different moments were clearly related to the increased concentration of released essential oil, as shown in Figure 9a. The first one, which was observed between *t* = 0 and *t* = 75 h, may have been related to the hydration of the outermost layer of the particles formed by gelatin. This data corroborates the rapid release rate (27 µg·mL^−1^·h^−1^) in the first hours. The gelatin layer (in contact with water in the release experiment) became more hydrated and turgid (in addition to being more solubilized than PCL), exposing the essential oil to the medium [113]. The fluorescence results confirmed the presence of some amount of essential oil within this part of the particle.

As observed, two different time intervals of increased concentration of released essential oil were clear and separated by a time interval of almost zero released concentration (between 50 h and 75 h). For this reason, we suggested that the first concentration of released essential oil (approximately 150 µg·mL^−1^) was due to the non-complexed essential oil. The proposition that some amount of the essential oil was encapsulated at this interface is supported by the second moment of controlled release, which was observed after 75 h: this second released concentration may have been related to the release of complexed essential oil observed between 75 h and 175 h. Then, a second increase of released essential oil concentration was observed. After 75 h of release, the complexed essential oil started to be released into the medium, resulting in approximately 150–200 µg·mL^−1^ (depending on pH). The release curves allowed obtaining new information about the design proposition of the developed particles. The release profile at all tested pHs suggested that part of the encapsulated essential oil (non-complexed) was released before and following the release of the complexed oil from 75 h. After 175 h, a considerable concentration of released essential oil was not observed.

**Figure 10:**
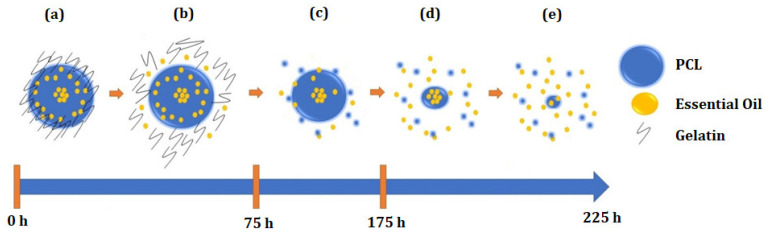
Schematic diagram of the speculative proposal for the interpretation of the controlled release associated with particle design as a function of time: (**a**) particle consisting mainly of gelatin, PCL, and essential oil, (**b**) hydration/solubilization process of the gelatin layer, allowing the release of some amount of essential oil, (**c**) starting of the degradation of PCL, (**d**,**e**) increasing of the released essential oil.

Figure 10 schematically shows a speculative proposal for the interpretation of the controlled release of the PNE system associated with the particle design [26,114]. Figure 10a shows a particle consisting mainly of gelatin, PCL, and essential oil, as well as the concentration of essential oil located within the particle.

Figure 10b suggests that, in the first hours (and under a high releasing rate), the gelatin layer underwent a hydration/solubilization process, allowing the release of some amount of essential oil. At this point, we can propose that the degradation of PCL also started (Figure 10c). Between 50 h and 75 h, the concentration of released essential oil remained practically zero, since part of the essential oil was complexed in the PCL matrix. Finally, from 75 h (Figure 10d), an increasing of the released essential oil was observed up to 225 h (Figure 10e).

Regarding the mathematical models of release proposed by Korsmeyer-Peppas and Higuchi [115], the association of the PNE system with these models was not determined. These models are applied to exponential or linear release curves [116]. Herein, the interesting pulsatile release mechanism may be useful in applications requiring different concentrations of essential oil in different time intervals [117,118].

## 4. Conclusions

The present research successfully developed a controlled release system based on polymeric carriers for the encapsulation of the essential oil from *P. nigrum*. The formulation stability was ensured based on the addition of preservatives, as well as on the sophisticated design allowed by the carriers, PCL and gelatin. The pulsatile release mechanism was achieved due to the complexation of some amount of essential oil in the PCL matrix. Active elements from medicinal plants are widely used by the population in general. In the encapsulated form, such bioactive molecules are greatly potentiated, despite the reduced concentration, and by the direct way they present themselves during the release process. Regarding the real viability as a product, we consider that the PNE system (and possibly the PTI) was the more stable system. The evaluation of several parameters such as EE%, pH, electrical conductivity, turbidity, and organoleptic properties allowed proposing their application, both as a biodefensive for controlling human disease vectors (such as larvae and mosquitoes of *Aedes aegypti* and *Anopheles aquasalis*) and for therapeutic purposes. Several reports have focused on the efficiency of *P. nigrum* essential oil in the control of a broad spectrum of agriculture pests. As an alternative controlling agent, the systems will favor a reduced number of reapplications, due to the pulsatile releasing mechanism. As a pharmaceutical product, when used internally or topically (and in safe concentrations), encapsulated essential oils can be of great value for the treatment of various diseases and mainly in the fortification and rehabilitation of patients. In order to have safe use, the development of a technical-scientific knowledge that guarantees the form of use and action mechanisms is necessary. Scientific studies seek to contribute to this evolution, and this is certainly the purpose of this study.

## Figures and Tables

**Table 1:** Average particle size measured by NTA, considering the unloaded and loaded systems with no added preservatives.

Parameters	Unloaded Particles	Loaded Particles
Mean (nm)	240 ± 8	224 ± 9
Mode (nm)	135 ± 3	122 ± 4
SD (nm)	174 ± 18	161 ± 16
D_10_ (nm)	118 ± 4	114 ± 3
D_50_ (nm)	162 ± 2	147 ± 4
D_90_ (nm)	519 ± 13	456 ± 11
Concentration (particles/mL)	(5.0 ± 0.3) × 10^10^	(6.0 ± 0.7) × 10^10^

Parameters D_10_, D_50_, and D_90_ indicate that 10%, 50%, or 90%, respectively, of the particle’s population presented a diameter of less than or equal to the specified value.

**Table 2:** Preliminary stability parameters obtained at (35 ± 2) °C for loaded particles containing preservatives.

**Parameters**	**PNE**		**PMI**		**PBS**	
Time (days)	0	15	0	15	0	15
EE (%)	98 ± 2	98 ± 2	98 ± 2	98 ± 2	98 ± 2	76 ± 3
Electrical Conductivity (µS·cm^−1^)	1043 ± 5	1050 ± 5	738 ± 5	900 ± 5	915 ± 5	1000 ± 10
Turbidity (NTU)	40 ± 1	45 ± 2	40 ± 1	40 ± 1	40 ± 1	60 ± 4
pH	8.00 ± 0.02	7.60 ± 0.03	8.00 ± 0.02	7.60 ± 0.03	8.00 ± 0.02	6.70 ± 0.04
Color	NA	NA	NA	NA	NA	SA
Odor	NA	NA	NA	NA	NA	SA
**Parameters**	**PED**		**PTI**		**PSC**	
Time (days)	0	15	0	15	0	15
EE (%)	98 ± 2	83 ± 3	98 ± 2	98 ± 2	98 ± 2	76 ± 3
Electrical Conductivity (µS.cm^−1^)	687 ± 5	1200 ± 10	856 ± 5	1100 ± 10	628 ± 5	5000 ± 10
Turbidity (NTU)	40 ± 1	50 ± 3	40 ± 1	80 ± 2	40 ± 1	70 ± 3
pH	8.00 ± 0.02	7.20 ± 0.03	8.00 ± 0.02	7.00 ± 0.04	8.00 ± 0.02	6.00 ± 0.03
Cor	NA	SA	NA	SA	NA	SA
Odor	NA	SA	NA	SA	NA	SA

EE (%) = Encapsulation Efficiency; NA = No Alteration; SA = Slight Alteration.

**Table 3:** Shelf-life stability of the PNE, PTI, and PSC systems at 25 °C evaluated every 30 days.

Systems	Time (Days)	EE (%)	Electrical Conductivity (µS·cm^−1^)	Turbidity (NTU)	pH	Color	Odor
**PNE**	0	(98 ± 2)	(1043 ± 2)	(40 ± 1)	(8.00 ± 0.02)	NA	NA
	30	(98 ± 2)	(1140 ± 3)	(40 ± 2)	(8.00 ± 0.03)	NA	NA
	60	(97 ± 1)	(1204 ± 4)	(42 ± 3)	(7.90 ± 0.04)	NA	NA
	90	(95 ± 2)	(1301 ± 3)	(45 ± 2)	(7.80 ± 0.06)	NA	NA
	120	(90 ± 2)	(1400 ± 4)	(45 ± 2)	(7.70 ± 0.06)	NA	NA
	150	(89 ± 2)	(1450 ± 3)	(46 ± 3)	(7.60 ± 0.07)	NA	NA
	180	(85 ± 2)	(1500 ± 3)	(48 ± 2)	(7.50 ± 0.08)	NA	NA
	**210**	**(84 ± 1)**	**(1532 ± 4)**	**(50 ± 2)**	**(7.40 ± 0.09)**	**NA**	**NA**
	240	(80 ± 1)	(1580 ± 6)	(53 ± 2)	(7.3 ± 0.1)	SA	SA
	270	(70 ± 1)	(1630 ± 7)	(55 ± 2)	(7.1 ± 0.1)	SA	SA
**PTI**	0	(98 ± 3)	(856 ± 2)	(40 ± 3)	(8.00 ± 0.02)	NA	NA
	30	(95 ± 2)	(1000 ± 4)	(41 ± 1)	(7.70 ± 0.02)	NA	NA
	60	(90 ± 4)	(1121 ± 4)	(44 ± 2)	(7.50 ± 0.04)	NA	NA
	90	(85 ± 4)	(1203 ± 5)	(47 ± 2)	(7.20 ± 0.04)	NA	NA
	**120**	**(80 ± 2)**	**(1342 ± 5)**	**(48 ± 1)**	**(7.00 ± 0.02)**	**NA**	**NA**
	150	(75 ± 2)	(1389 ± 6)	(50 ± 3)	(6.80 ± 0.03)	SA	SA
	180	(72 ± 2)	(1409 ± 7)	(55 ± 4)	(6.50 ± 0.03)	SA	SA
	210	(70 ± 2)	(1500 ± 10)	(60 ± 5)	(5.90 ± 0.03)	SA	SA
**PSC**	**0**	**(98 ± 2)**	**(800 ± 2)**	**(40 ± 3)**	**(8.00 ± 0.03)**	**NA**	**NA**
	30	(90 ± 2)	(1100 ± 2)	(50 ± 7)	(7.00 ± 0.03)	SA	SA
	60	(70 ± 2)	(1500 ± 9)	(60 ± 5)	(5.40 ± 0.03)	SA	SA

NA = No Alteration; SA = Slight Alteration.

**Table 4:** Shelf-life stability of the PNE, PTI, and PSC systems at 35 °C evaluated every 30 days.

Systems	Time (Days)	EE (%)	Electrical Conductivity (µS·cm^−1^)	Turbidity (NTU)	pH	Color	Odor
**PNE**	0	(98 ± 2)	(1043 ± 5)	(40 ± 1)	(8.00 ± 0.02)	NA	NA
	30	(90 ± 2)	(1043 ± 4)	(42 ± 3)	(7.90 ± 0.03)	NA	NA
	60	(78 ± 2)	(1060 ± 4)	(45 ± 3)	(7.70 ± 0.06)	NA	NA
	**90**	**(73 ± 2)**	**(1070 ± 3)**	**(49 ± 2)**	**(7.00 ± 0.08)**	**NA**	**NA**
	120	(70 ± 2)	(1080 ± 5)	(50 ± 3)	(6.50 ± 0.05)	SA	SA
**PTI**	**0**	**(98 ± 2)**	**(856 ± 2)**	**(40 ± 3)**	**(8.00 ± 0.02)**	**NA**	**NA**
	30	(70 ± 4)	(1621 ± 4)	(70 ± 2)	(5.00 ± 0.04)	IA	IA
**PSC**	**0**	**(98 ± 2)**	**(800 ± 2)**	**(40 ± 3)**	**(8.00 ± 0.03)**	**NA**	**NA**
	30	(50 ± 2)	(1550 ± 4)	(70 ± 7)	(5.00 ± 0.03)	IA	IA

NA = No Alteration; SA = Slight Alteration; IA: Intensive Alteration.

## Data Availability

Not applicable.
